# Long-Term Effects of Multimedia Education and “Foot Alerts” Through the MyU App on Diabetic Foot Care in Individuals With Moderate-High Risk: Pilot Randomized Controlled Trial

**DOI:** 10.2196/78261

**Published:** 2026-01-13

**Authors:** Ameenah Alawadhi, Kay Scarsbrook Khan, Grace Messenger, Alice Cusworth, Mohammad Assi, Stuart R Gray, Ebaa Al-Ozairi

**Affiliations:** 1 Clinical Care Research and Trials Unit, Dasman Diabetes Institute, P.O. Box 1180, Dasman 15462, Kuwait City, 1180, Kuwait, 965 22242999 ext 3111; 2 Podiatry Unit, Dasman Diabetes Institute, Kuwait City, Kuwait; 3 Podiatry Unit, Harrogate and District NHS Foundation Trust, North Yorkshire, United Kingdom; 4 Podiatry Unit, North Cumbria Integrated Care NHS Foundation Trust, Cumbria, United Kingdom; 5 Technology Department, Dasman Diabetes Institute, Kuwait City, Kuwait; 6 School of Cardiovascular and Metabolic Health, University of Glasgow, Glasgow, United Kingdom

**Keywords:** behavior, DFU, diabetic foot ulceration, foot care, multimedia education, smartphone

## Abstract

**Background:**

Diabetic foot ulceration (DFU) is the leading cause of nontraumatic amputations in people with diabetes. Research shows that improving patient awareness can result in short-term improvements, but Cochrane reviews report insufficient high-quality evidence.

**Objective:**

This study aims to investigate the effects of multimedia presentation and smartphone alerts to enhance long-term knowledge and foot care behaviors in individuals at moderate-to-high risk of DFU.

**Methods:**

Participants were randomized to a control group, receiving usual diabetic foot care advice (n=40), or an intervention group, receiving a multimedia diabetic foot care presentation and regular “foot alerts” through the MyU smartphone app on top of usual care (n=37). Patient’s knowledge and behaviors related to diabetic foot care were assessed at baseline and after 12 months. Repeated measures ANOVA was conducted in both intention-to-treat and per-protocol analyses to evaluate the intervention’s effectiveness.

**Results:**

The findings were consistent across intention-to-treat and per-protocol analyses. In the intervention group, the number of podiatry visits was positively correlated with improved foot care behavior (*r*=0.408; *P*=.02), while the control group showed a negative correlation (*r*=−0.402; *P*=.02). No significant correlations were observed with knowledge scores. Although no significant time×group interactions were seen, the main effects of time were found for both knowledge (η²=0.12; *P*=.004) and behavior scores (η²=0.31; *P*<.001). Post hoc analysis showed a decline in knowledge scores in the control group (Cohen *d*=−0.24; *P*=.007) and improvements in behavior scores in both groups (Cohen *d*: intervention=0.61, control=0.63; all *P*<.001).

**Conclusions:**

The MyU app−based multimedia intervention was associated with improved foot care behaviors over 12 months, indicating potential benefits as an adjunct to usual diabetic foot care. However, no significant changes in diabetic foot care knowledge were observed. These findings suggest that while the applied digital multimedia tool may support behavior change, further research is needed to enhance knowledge retention and clinical impact. The study revealed that multimedia education alone may not be effective for long-term improvement in foot self-care knowledge and behavior among individuals at moderate-high DFU risk, but the reinforcement of educational material during follow-up podiatry visits could be effective.

## Introduction

The escalating incidence of diabetes worldwide has turned it into a global pandemic impacting over 500 million individuals [1]. Health costs associated with diabetes are expected to reach US $1054 billion by 2045, up from US $966 billion in 2021 [2], with 80% of this spent on managing avoidable complications [3]. One of the most common long-term complications of diabetes is diabetic foot ulceration (DFU), with a lifetime risk of developing a DFU of between 12% and 25% [4-6]. DFU continues to be the leading cause of nontraumatic lower limb amputation [7], accounting for 84% of lower limb amputations [8]. Studies have shown that people with diabetes are 10‐30 times more likely to have a lower limb amputation than someone without diabetes [9,10]. Furthermore, Armstrong et al [11] reported a 5-year mortality rate in people with DFU of as much as 43% at 1 year, worse than some of the most common cancers, and postamputation rates have shown that only 50% of patients survive the first year following amputation [12].

The early detection and treatment of minor foot injuries have been shown to reduce amputation rates by between 49% and 85% [13], yet amputation rates continue to rise [14]. Improved patient awareness and correct foot self-care practices can interrupt the amputation pathway [6]. Although systematic reviews have demonstrated the efficacy of foot care education in enhancing self-care behaviors and knowledge [15-17], there are insufficient data to support its clinical use in reducing ulceration and amputation incidence [18,19]. Furthermore, the delivery of education sessions is particularly difficult for those with limited literacy, and debates are ongoing about whether video-based or printed materials are most effective [20,21]. Comprehensive foot care education, which encompasses assessment, discussion, counseling, home visits, and telephone calls, has been proposed to enhance diabetic foot care and educational programs [18]. However, a systematic review of 6 randomized controlled trials (RCTs) revealed insufficient evidence to support its efficacy [22]. More recently, it has been suggested that mobile apps can effectively prevent DFU recurrence [23] and improve diabetic foot care outcomes by incorporating at least 1 information communication tool [24]. However, many of the RCTs focused on patients at low risk, limiting their utility to support improved clinical outcome in the measure of reducing the rate of ulceration [18,19,22].

Given these findings and the estimated financial savings in the prevention of DFU, there is a need to focus on its prevention [25]. This study, therefore, aims to (1) establish if the use of a multimedia presentation and weekly alerts from a smartphone app as an education tool improves long-term knowledge and foot care behaviors of patients with diabetes when compared to usual care, (2) assess the association between the change in participants' knowledge and behavior and the number of podiatry appointments over the study period, and (3) compare the number of new DFUs and hemoglobin A_1c_ (HbA_1c_) levels between the 2 study groups.

## Methods

### Study Design

This randomized, investigator-blinded, 2-armed, pilot study was conducted on patients with diabetes who visited Dasman Diabetes Institute (Kuwait City, Kuwait) clinics from January 2019 to May 2024. Each participant was fully informed about the study prior to giving their written informed consent.

### Eligibility Criteria

Participants had to meet the following inclusion criteria: (1) adult patients (≥18 years of age) with type 2 diabetes or adults with type 1 diabetes for ≥5 years, (2) be of medium or high DFU risk, defined as having at least 1 diabetic foot risk factor (loss of pain perception, peripheral vascular disease, foot deformity, history of DFU, or amputation), (3) able to understand study procedures and comply with them for the entire length of the study, and (4) must own a smartphone with internet access and agree to have the phone app uploaded for the duration of the study. People with chronic kidney disease, cognitive impairment, active psychiatric illness, inability to give written informed consent, hearing or visual impairment, or phone app inaccessibility for more than 4 weeks were excluded from the study.

### Procedures

#### Overview

The study consisted of 2 main visits (at baseline and 12 mo) and up to 6 interim follow-up visits during the 1-year study period. A total of 98 participants were randomized by an independent researcher to either the control (“Usual Care” section) or intervention (“Multimedia Educational Intervention” section) arm in a 1:1 ratio. Participants were instructed not to disclose any information about their assigned group to the research team to maintain confidentiality. All written information for this study went through a process of back translation from English to Arabic, focusing on conceptual rather than literal translations and using natural language for a broad audience [26]. Prior to the intervention period, participants underwent diabetic foot assessment and were classified based on their diabetic foot risk as per NICE (National Institute for Health and Care Excellence) guidelines [27], with participants with moderate risk attending 2 additional follow-up visits at 6-month intervals and participants with high risk attending 6 additional follow-up visits at 2-month intervals. At the baseline visit, HbA_1c_ was measured, and participants received their assigned diabetic foot care education route. Then, they were asked to complete a diabetic foot care knowledge questionnaire and a foot care and diabetes self-care behaviors questionnaire. Interim follow-up visits included a visual inspection of feet, verbal advice in the usual manner (for both groups), and an educational audio-visual prerecorded presentation (for intervention group only). At the 12-month visit, all participants completed a foot care knowledge questionnaire, a foot care and diabetes self-care behaviors questionnaire, and underwent an HbA_1c_ test.

#### Usual Care

The control group received usual care and the usual route of education with educational leaflets to take home. Participants received verbal advice during their podiatry appointment about daily foot inspections, wearing well-fitting shoes, what to do in an emergency, and the use of emollient. More specific advice was given depending on the participant’s risk classification. This usual route involved information by the podiatrist translated by a bilingual but not native Arabic-speaking nurse.

#### Multimedia Educational Intervention

**Figure 1.**
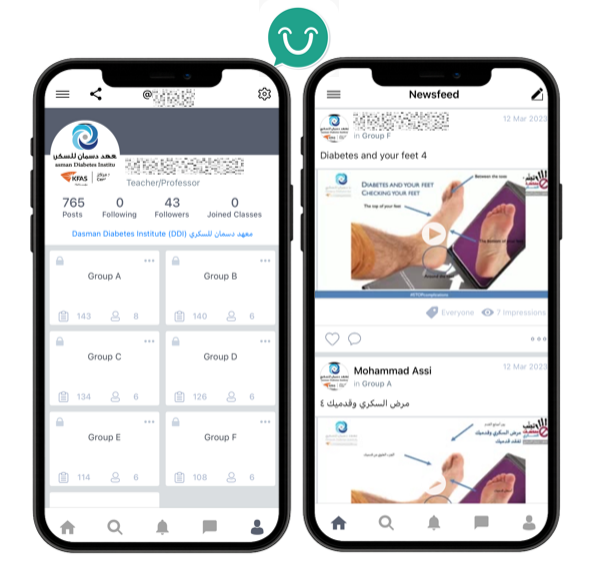
MyU app interface showing the layout and key features used to deliver educational content. The home screen provides access to 12 educational videos, which were assigned to participants according to their recruitment date. Weekly push notifications (shown at the bottom of the screen) prompted users to engage with the app and view the assigned videos displayed on the Newsfeed page.

The intervention group received an educational presentation and a smartphone app providing weekly foot alerts for the duration of the study, in addition to the usual care, education route, and leaflet. The details of the MyU app interface, including the layout and key features used to deliver educational content, are illustrated in Figure 1. The educational presentation was a 10-minute audio-visual prerecorded presentation based on the internationally recognized advice on preventing diabetic foot complications including good diabetes control, daily foot inspections, and footwear advice [27-29]. The research podiatry clinic allowed only 1 participant to attend at a time, ensuring privacy for viewing the educational video. In addition, a teaching- or education-based smartphone app called MyU was installed onto their smartphones, allowing educational information to be uploaded and delivered remotely. The app also remotely recorded the number of times the content was accessed by each participant. Adherence to the intervention was monitored based on these access logs, where each “access” was defined as opening the app and viewing the educational content. However, more detailed engagement metrics such as video completion or time spent on content were not captured. Adherence (%) was calculated as the percentage of scheduled videos viewed, based on the total video views recorded by the MyU app for each participant. Both the absolute number of videos viewed (count) and the corresponding percentage adherence were reported. This app also delivered a weekly “foot-alert” (notification) to remind the participant to view the uploaded educational video. There were 12 unique educational videos “alerts” repeated at 3-month cycles. Repeating the educational method has been shown to improve health literacy [30]. All foot alerts were uploaded in both Arabic and English.

### Foot Care Knowledge Questionnaire

The foot care knowledge questionnaire used in this study has been adapted from Pollock et al [31] and Rheeder et al [32]. The questionnaire covers 2 sections. The first section consisted of 7 questions with 3 options each: correct, incorrect, and don’t know. A correct answer was awarded 1 point, while incorrect or “don’t know” responses received 0 points. The second section consisted of 5 questions with multiple-choice options. A correct answer was awarded 1 point, while an incorrect answer received 0 points. The maximal total score is 12 points, with higher scores indicating a better understanding of foot care knowledge.

### Foot Care and Diabetes Self-Care Behaviors Questionnaire

The foot care and diabetes self-care behaviors questionnaire has been adopted from the Summary of Diabetes Self-Care Activities (SDSCA). The SDSCA has been found to have both reliability and validity as a standard measure of diabetes self-management [33]. Furthermore, this test has been shown to be reliable and valid, giving consistent results when translated into Arabic in a sample size of 243 participants [34]. The SDSCA scale measures the frequency of each self-care activity in the last 7 days for 4 aspects related to diabetes routine: foot care (8 scaled questions), blood-glucose testing (2 scaled questions), medications adherence (2 scaled questions), and smoking habits (2 questions). For this analysis, the score of the scaled questions (from 0 to 7 d) was calculated as the average of responses within each section [33]. A higher total score indicates better foot self-care behavior.

### Sample Size

Although this is a pilot study, a sample size of 98 with a 1:1 ratio between intervention and control group (49:49) was calculated using G*power software (version 3.9.1.2; Heinrich-Heine-Universität Düsseldorf). An a priori difference between 2 independent means tests was performed to find a significance interaction with a power of 80% and probability of type 1 error of 0.05. The effect from a study by Baba et al [35] was a significant mean change of 1.8 (SD 2.6) reduction in foot score (based on the presence and severity of 15 podiatry disorders; n=78) compared to a 0.1 (SD 2.6) reduction in the foot score after receiving interactive education (n=76) over 3 months (effect size=0.58).

### Statistical Analysis

Data analysis was performed using SPSS Statistics (version 29.0; IBM Corp). Descriptive statistics were represented as mean (SD) for continuous variables and as frequencies for categorical variables. The Shapiro-Wilk test was used to assess normality for each continuous variable to determine the appropriate use of parametric or nonparametric tests. Chi-square tests and independent 2-tailed *t* tests were conducted to test to examine baseline differences between the groups and to compare the frequency of new DFU cases between the study groups. The Wilcoxon signed-rank test was used when the assumption of normality was violated.

Both intention-to-treat (ITT) and per-protocol (PP) analyses were conducted. The ITT analysis included all randomized participants with the available outcome data, regardless of adherence or dropout status. A linear mixed model was used to evaluate group, time, and group×time interaction effects on the outcome measures: foot care knowledge scores, foot care, blood-glucose testing, and medication adherence behaviors.

Post hoc pairwise comparisons were conducted using the matched pair 2-tailed Student *t* test or Wilcoxon signed-rank test, with Bonferroni adjustment applied for multiple comparisons. Bivariate Pearson or Spearman correlation analyses were performed to evaluate the strength and direction of relationships between the change in foot care knowledge scores and foot self-care behavior: scores and the number of follow-up visits. Changes in scores were calculated as 12 month values − baseline values. *P* values <.05 were considered statistically significant.

### Ethical Considerations

Ethical approval was granted by the Dasman Diabetes Institute Ethical Review Committee (RA HM‑2018‑044). The study was conducted in accordance with the Declaration of Helsinki and Good Clinical Practice guidelines and was registered at ClinicalTrials.gov (NCT03934944). The trial was conducted and reported in accordance with the CONSORT-EHEALTH (Consolidated Standards of Reporting Trials of Electronic and Mobile Health Applications and Online Telehealth) 2011 guidelines (Checklist 1).

Written informed consent was obtained from all participants prior to enrollment. Participant privacy and confidentiality were strictly maintained throughout the study: all data were deidentified and stored on secure, password-protected servers accessible only to authorized research personnel, and no personally identifiable information was included in the analysis or reported in the manuscript. Participants did not receive financial compensation for their participation.

## Results

**Figure 2.**
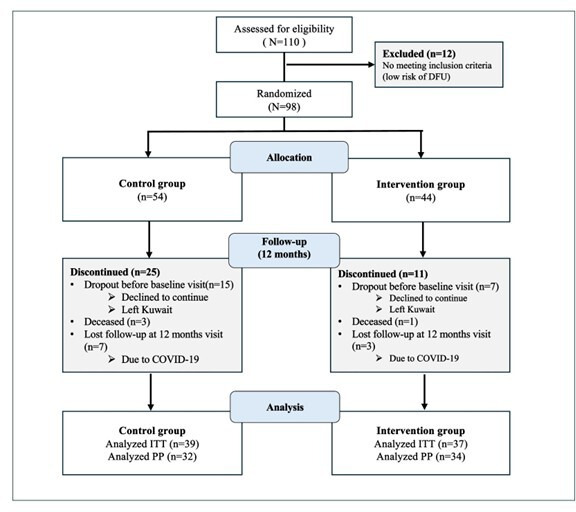
Participants flow chart. DFU: diabetic foot ulceration; ITT: intention-to-treat; PP: per-protocol.

A total of 66 participants completed the study visits and were included in the final PP analysis (Figure 2). Additionally, the ITT analysis included 77 participants who were randomized and had at least 1 outcome measure available. Table 1 shows the baseline characteristics of study participants in both ITT and PP populations. In the ITT population, the mean age of the participants was 60 (SD 7.85) years in the intervention group and 62 (SD 9.31) years in the control group. In both groups, the majority of the study participants were male participants, had attained an undergraduate degree, were nonsmokers, and had type 2 diabetes for over 20 years. In the intervention group, 75.7% (n=28) were classified as having a high DFU risk compared to a 62.5% (n=20) in the control group. All baseline characteristics were statistically comparable between the groups in both ITT and PP analyses, except for BMI, which showed a statistically significant difference between the groups in the ITT sample (*P*=.01) but not in the PP sample. Dropout analysis revealed that 8 participants from the control group (6 with high DFU risk and 2 with moderate risk) and 3 participants from the intervention group (all high risk) discontinued the study.

**Table 1.** General characteristics according to study group and analysis carried out.

Variable	ITT ^a^			PP ^b^		
	Intervention ( n = 37 )	Control ( n = 40 )	*P* value	Intervention ( n = 34 )	Control ( n = 32 )	*P* value
Age (y), mean ( SD )	60.43 (7.85)	62.39 (9.81)	.34	61.06 (7.87)	62.70 (9.41)	.44
BMI (kg/m ^2^ ), mean ( SD )	34.05 (5.69)	36.94 (3.2)	.01 ^c^	34.05 (5.69)	32.09 (6.04)	.18
Sex, n (%)			.83			.65
Male	24 (65)	25 (63)		21 (62)	20 (63)	
Female	13 (35)	15 (38)		13 (38)	12 (38)	
Ethnicity, n (%)			.57			.49
Arabic	2 (5)	3 (8)		2 (6)	3 (9)	
Asian	0 (0)	1 (3)		0 (0)	1 (3)	
Kuwaiti	35 (95)	36 (90)		32 (94)	28 (88)	
Education l evel, n (%)			.23			.21
Junior school	8 (22)	2 (5)		6 (18)	1 (3)	
Senior school	9 (24)	11 (28)		9 (27)	7 (23)	
Undergraduate	14 (38)	22 (55)		14 (41)	19 (61)	
Postgraduate	7 (19)	4 (10)		5 (15)	4 (13)	
Smoking s tatus, n (%)			.51			.21
Smoker	6 (16)	5 (13)		6 (18)	3 (9)	
Nonsmoker	26 (65)	23 (62)		26 (81)	21 (62)	
Ex-smoker	7 (21)	5 (13)		7 (21)	3 (9)	
Type of d iabetes, n (%)			.58			.33
Type 1	3 (8)	2 (5)		3 (9)	1 (3)	
Type 2	34 (92)	38 (95)		31 (97)	31 (91)	
Duration of d iabetes (y), n (%)			.82			.83
0‐9	4 (11)	5 (13)		4 (13)	3 (9)	
10‐19	11 (30)	14 (35)		9 (28)	11 (34)	
>20	22 (60)	21 (53)		19 (59)	18 (56)	
HbA_1c_ ^d^, mean ( SD )	8.32 (1.87)	8.14 (1.61)	.65	8.29 (2.04)	8.17 (1.68)	.61
Overall ABPI ^e^, mean ( SD )	1.17 (0.13)	1.18 (0.07)	.95	1.186 (0.21)	1.13 (0.14)	.56
Average VPT ^f^, mean ( SD )	52.60 (118.3)	50.86 (113.7)	.94	53.43 (123.55)	55.84 (129.16)	.94
Risk f actors, n (%)						
PAD^g^	7 (19)	6 (15)	.57	6 (18)	5 (16)	.82
LOPS^h^	33 (89)	37 (93)	.41	30 (88)	30 (92)	.43
Foot deformity	22 (60)	19 (48)	.13	21 (62)	16 (50)	.33
History of DFU^i^	18 (49)	16 (40)	.5	16 (47)	13 (41)	.59
History of amputation	7 (19)	8 (20)	.61	7 (21)	7 (22)	.89
History of Charcot	5 (14)	4 (10)	.56	4 (12)	3 (9)	.75
DFU risk classification, n (%)			.12			.13
High	28 (76)	25 (63)		25 (76)	18 (58)	
Moderate	9 (24)	15 (38)		9 (24)	14 (42)	

a ITT: intention-to-treat.

b PP: per protocol.

c *P value <.05

d HbA_1c_: hemoglobin A_1c_.

e ABPI: ankle-brachial pressure index.

f VPT: vibration perception threshold.

g PAD: peripheral artery disease.

h LOPS: diabetic peripheral neuropathy with loss of protective sensation.

i DFU: diabetic foot ulceration.

**Figure 3.**
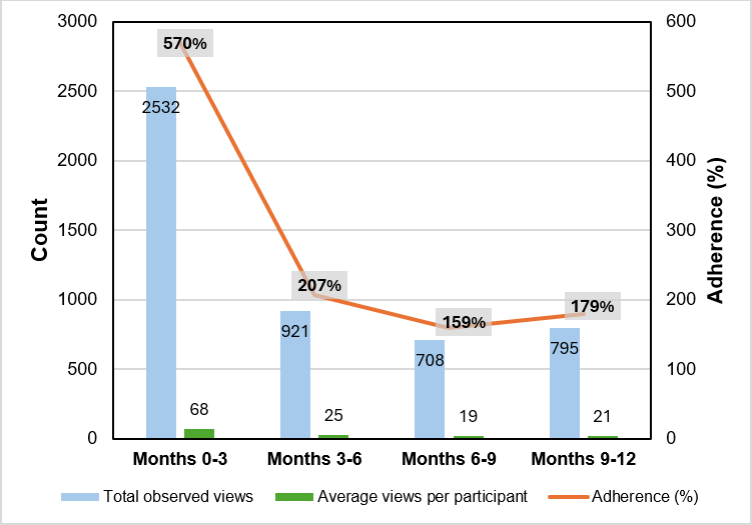
Total video views (blue bars), average views per participant (green bars), and adherence percentage (line) across study time points, based on MyU app data. Values represent group-level engagement with 12 videos per interval. Adherence (%) represents the percentage of the scheduled videos viewed, calculated from the total video views recorded by the MyU app. Both the absolute number of videos viewed (count) and the corresponding percentage are shown.

Adherence was estimated using the total video views recorded by the MyU app. The actual views exceeded the maximum expected views per interval (444 views), especially at baseline (mo 0‐3: 2532 views, 570%), indicating repeated viewing. Engagement declined over time (mo 9‐12: 795 views, 179%). These counts serve as a group-level proxy for adherence (Figure 3).

Both ITT and PP analyses revealed no significant time×group interactions for diabetic foot care knowledge and behavior scores. However, there were significant main effects of time for both outcomes (ITT: *F*_1,70_=6.98, *P*=.01, partial η²=0.09 for knowledge; *F*_1,70_=28.36, *P*<.001, partial η²=0.29 for behavior; PP: *F*_1,64_=8.760; *P*=.004, partial η²=0.12 for knowledge scores; *F*_1,64_=28.139; *P*<.001, partial η²=0.31 for behavior scores). Post hoc within-group comparisons showed a significant reduction in diabetic foot care knowledge scores (ITT: mean difference −0.878, SE 0.366 points, *P* value=.02, Cohen *d=*−0.21; PP: mean difference −1, SE 0.357 points, *P* value=.007, Cohen *d=*−0.22) in the control group, with no change in the intervention group (ITT: mean difference −0.45, SE 0.344 points, *P*=.2, Cohen *d=*−0.38; PP: mean difference −0.47, SE 0.346 points, *P*_*t* test_=.18, Cohen *d=*−0.48). For diabetic foot care behavior, post hoc within-group comparisons showed a significant increase in both control (ITT: mean difference 1.129, SE 0.29 points, *P*<.001, Cohen *d=*0.62*;* PP: mean difference 1.109, SE 0.3 points, *P*<.001, Cohen *d*=0.61) and intervention (ITT: mean difference 1.139, SE 0.3 points, *P*<.001, Cohen *d=*0.60; PP: mean difference 1.121, SE 0.29 points, *P*<.001, Cohen *d=*0.63) groups. No interaction or main effects were seen in other self-care behaviors scores such as blood-glucose testing and medication adherence (Tables2  3).

**Table 2.** Intention-to-treat (ITT) analysis: comparison of knowledge and behavior of diabetic foot self-care between the groups before and after the intervention (n=77; intervention group n=37 and control group n=40).

Group	Baseline, estimated mean (SE)	12 months, estimated mean (SE)	Within-group comparison, *P* value (Cohen *d*)	Time effect, *P* value (η²)	Group effect, *P* value (η²)	Interaction, *P* value (η²)
Knowledge of diabetic foot care score						
Intervention	6.51 (0.31)	6.10 (0.29)	.2 (–0.38)	.01^a^ (0.09)	.42 (0.01)	.39 (0.01)
Control	6.42 (0.27)	5.54 (0.36)	.02^a^ (–0.21)	—^b^	—	—
Foot care and diabetes self-care behaviors						
Foot-care score						
Intervention	3.49 (0.19)	4.64 (0.36)	.001^a^ (0.60)	<.001^a^ (0.29)	.12 (0.04)	.98 (<0.001)
Control	3.04 (0.17)	4.16 (0.36)	.001^a^ (0.62)	—	—	—
Blood-glucose testing score						
Intervention	4.77 (0.43)	5.16 (0.48)	.45 (–0.07)	.06 (0.05)	.07 (0.001)	.41 (0.009)
Control	4.30 (0.42)	5.34 (0.42)	.08 (0.03)	—	—	—
Medication adherence score						
Intervention	5.50 (0.35)	5.63 (0.42)	.83 (0.29)	.92 (0.0001)	.83 (0.0006)	.67 (0.002)
Control	5.60 (0.38)	5.38 (0.38)	.68 (0.12)	—	—	—

a *P*<.05.

b Em dashes indicate values that are identical for both groups because these results represent overall ANOVA model effects rather than group‑specific estimates.

**Table 3.** Per-protocol (PP) analysis: comparison of knowledge and behavior of diabetic foot self-care between the groups before and after the intervention (n=66; intervention group n=34 and control group n=32).

Group	Baseline, estimated mean (SE)	12 months, estimated mean (SE)	Within-group comparison, *P* value (Cohen *d*)	Time effect, *P* value (η²)	Group effect, *P* value (η²)	Interaction, *P* value (η²)
Knowledge of diabetic foot care score						
Intervention	6.62 (1.87)	6.15 (1.74)	.18 (–0.48)	.004^a^ (0.12)	.49 (0.007)	.29 (0.017)
Control	6.63 (1.75)	5.63 (1.87)	.007^a^ (–0.24)	—^b^	—	—
Foot care and diabetes self-care behaviors						
Foot-care score						
Intervention	3.51 (1.21)	4.63 (2.2)	<.001^a^ (0.63)	<.001^a^ (0.31)	.13 (0.034)	.97 (<0.001)
Control	3.03 (0.98)	4.14 (1.55)	<.001^a^ (0.61)	—	—	—
Blood-glucose testing score						
Intervention	4.66 (2.71)	5.07 (2.91)	.41 (–0.07)	.09 (0.043)	.91 (0.0002)	.49 (0.01)
Control	4.3 (2.75)	5.29 (2.46)	.13 (–0.07)	—	—	—
Medication adherence score						
Intervention	5.35 (2.32)	5.61 (2.51)	.66 (–0.26)	.98 (<0.001)	.68 (0.003)	.79 (0.001)
Control	5.46 (2.54)	5.34 (2.26)	.71 (–0.15)	—	—	—

a *P*<.05.

b Em dashes indicate values that are identical for both groups because these results represent overall ANOVA model effects rather than group‑specific estimates.

**Figure 4.**
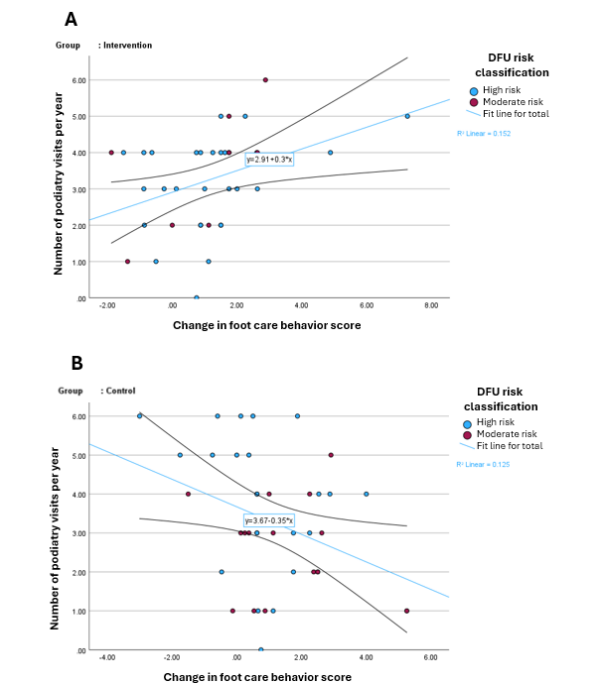
Association between the number of podiatry visits and the change in diabetic foot care behavior in the intervention group (**A**) and control group (**B**). DFU: diabetic foot ulceration.

The mean of the number of podiatry follow-up visits over the 12 months was comparable between the ITT group (control: mean 3.27, SD 1.7 vs intervention: mean 3.24, SD 1.3 visits; *P*=.93) and PP group (control: mean 3.3, SD 1.6 vs intervention: mean 3.26, SD 1.3 visits; *P*=.83). Similarly, among participants at a high DFU risk, those in the intervention attended mean 3.52 (SD 1.2; ITT) and mean 3.64 (SD 1.1; PP) visits, while those in the control group attended mean 3.72 (SD 1.6; ITT) and mean 3.77 (SD 1.6; PP) visits. Among participants at a moderate DFU risk, the intervention group attended a mean of 2.25 (SD 1.48; ITT) visits, in both ITT and PP analyses, while the control group attended a mean of 2.53 (SD 1.6; ITT) visits and a mean of 2.76 (SD 1.58; PP) visits. The number of podiatry visits, however, was positively associated with the change in foot care behavior in the intervention group (ITT: *r*=0.390, *P*=.02; PP: *r*=0.408, *P*=.02), while the control group showed a negative correlation (ITT: *r*=−0.354, *P*=.03; PP: *r*=−0.402, *P*=.02; Figure 4). In ITT analysis, a significant negative association was observed in the control group between the number of podiatry visits and the change in the knowledge scores (*r*=−0.357; *P*=.02), but no such association was found in the intervention group. In the PP analysis, no significant associations were observed in either group. The frequency of new DFU during the course of the study was comparable between the 2 groups (ITT: control=17.5% vs intervention=21.6%, *P*=.64; PP: control=18.8% vs intervention=17.6%, *P*=.91). Both ITT and PP analyses revealed no statistically significant difference in the HbA_1c_ at 12 months between the intervention (ITT: mean 7.86%, SD 0.38%; PP: mean 7.93%, SD 1.08%) and the control (ITT: mean 8.28%, SD 0.36%; PP: mean 8.45%, SD 1.4%) groups, with a mean difference of 0.58% (95% CI −0.93 to 2.09; *P*=.44) in ITT, and 0.52% (95% CI −0.14 to 1.19; *P*=.12) in the PP analyses.

## Discussion

### Principal Findings

This pilot study investigated the effectiveness of a multimedia educational approach in improving foot care knowledge and behavior among individuals with diabetes over a 12-month period. Overall, this study found that there was no long-term benefit to using multimedia educational tools in addition to standard usual podiatry care to improve participants’ knowledge and behavior. However, post hoc comparisons showed that while both usual care and multimedia educational approaches enhanced the foot care behaviors of participants, the usual care settings resulted in a decline in their foot care knowledge, whereas the multimedia educational settings maintained it. The effect on glycemic control, however, was marginal and not clinically relevant (nonsignificant reduction in HbA_1c_ of only 0.52%). The consistency of results between the ITT and PP analyses supports the robustness of our findings and indicates that participant dropout did not substantially bias the outcomes. This consistency increases confidence that the observed effects are likely to be representative of real-world settings.

Delivering the appropriate foot self-care education is a crucial strategy for reducing the risk of DFUs in people with diabetes [36]. Though there is currently no consensus regarding the optimal education method on diabetic foot care among health care professionals [37]. In light of technological advancements, several interventional studies using smartphone apps and media aids were conducted to educate patients about foot self-care [38]. But their effectiveness remains unclear. To the best of our knowledge, this is the first study that examined the long-term (12 mo) effects of multimedia educational tools on diabetic foot care including both audio-visual presentation and smartphone app. In support of our findings, a short-term (3 mo) RCT on 120 individuals with diabetes found that an audio-visual educational presentation, supplementary to standard diabetic care and regular reinforcement of education in outpatient clinic, significantly enhanced knowledge scores by +1.17 (95% CI 0.7‐1.64, *P* value<.001) compared to those receiving standard care alone (+0.1, 95% CI −0.3 to 0.5, *P*=.62), whereas the practice scores were significantly improved in both groups (intervention: +1.6, 95% CI 1.09‐2.11, *P*<.001; control: +0.48, 95% CI 0.16‐0.8, *P*=.004) [39]. A 3-week educational program involving presentation slides, booklets, and group discussions significantly improved knowledge (intervention: mean 8.08, SD 0.88 vs control: mean 7.17, SD 1.91; *P*=.02) and behavior scores (intervention: mean 19.32, SD 6.48 vs control: mean 18.96, SD 7.36; *P*=.0001) compared to usual care [40].

Contrary to our findings regarding the use of a smartphone app, a recent RCT study involving 88 participants found that m-DAKBAS, an educational mobile diabetic foot care app that involved information, prevention, and management interfaces, significantly improved knowledge scores over a 6-month period (intervention: mean 16.83, SD 1.56 vs control: mean 15.05, SD 2.17; *P*=.0001), with no significant differences in behavior scores (intervention: mean 62.59, SD 7.76 vs control: mean 59.45, SD 10.53*; P*=.23) [41]. Furthermore, a 2-month use of the MobileDiabetes self-care app, which allowed patients to improve their self-care practices with flexibility in timing, location, and choices, has been shown to increase both patients’ self-care knowledge and behavior by 17% and 22%, respectively [42]. In a 1-month RCT, an animation-supported app (Mobile Diabetic Foot Care Education; M-DFCE) that delivered 2 push alerts per week and included a cartoon animation providing basic education on daily foot care to prevent foot wounds was evaluated [43]. The experimental group outperformed the control group in terms of foot care behavior (mean difference: 11.28, SD 10.47 for intervention vs 0.6, SD 24.85 for control; *P* =.01) and knowledge (mean difference: 0.87, SD 1.21 for intervention vs 0.01, SD 1.25 for control; *P*=.002) when compared to baseline [43]. According to a recent systematic review, for patients with diabetes, using a mobile health app enhanced their awareness of the disease and their capacity for self-care in studies of durations of less than 6 months [44]. A study involving 58 patients with uncontrolled diabetes found that integrating self-management, through peer-supporting video, a quiz game, and a feedback system, with the Diabetes Care App for 5 weeks, improved foot care behavior significantly (*P*<.01) when compared to usual care [45]. Similar improvements in foot care practices were observed on patients with type 2 diabetes after 1 month [46] and 3 months [47] of using self-management integration with smartphone apps. Furthermore, the use of smartphone apps and alerts interventions has been associated with improved glycemic control [48], which is contrary to our findings. We found a minor and comparable decrease in HbA_1c_ among the 2 groups, which might be reflected by their scores of the blood glucose-testing and medication adherence. While multimedia education on foot self-care is essential for improving the knowledge and behavior of individuals at a moderate-to-high risk of DFUs, it did not have a significant impact on glycemic control or long-term behavior changes.

In this study, there was no discernible difference in the overall incidence of DFU across the groups, although the overall incidence and thus the study power were low. This may suggest that the use of multimedia aids in foot self-care does not offer additional benefits above the usual care education. In agreement with our findings, a systematic review of 6 RCTs found insufficient evidence for the benefit of an integrated care approach, which involved combining multiple DFU prevention strategies at different levels of care (including the patient, health care provider, and health care structure), indicating the need for more high-quality studies [22]. Another systematic review of 11 RCTs, on the other hand, showed that educational technologies were protective against the incidence of lower limb amputations (relative risk=0.53, 95% CI 0.31‐0.90; *P*=.02) and DFU (relative risk=0.40, 95% CI 0.18‐0.90; *P*=.03), despite the lack of evidence of certainty assessment [49]. Other studies suggested that a more focused and intensive educational approach should be adopted to reduce the incidence of DFU. An RCT on people with type 2 diabetes found that 2-hour focused education sessions, including practical exercises on foot care behaviors, were effective in preventing the incidence of DFU during 6-month follow-ups compared to the control group (incidence of DFU=0% vs 10%; *P*=.01) [50]. Similarly, a quasi-experimental study found that combining educational sessions with foot assessment and care reduced the recurrence rate of DFU to 13.3% compared to 33.3% in the control group [51]. Intensive education approaches that included training and customized footwear have been shown to reduce the incidence rate of new DFU compared to usual care by 18% and 31%, respectively [52]. The results from a systematic review and meta-analysis of RCTs demonstrated that, in comparison to the control group, an intensive educational approach—a 45- to 1-hour education session reinforced by written instructions—significantly decreased the risk of DFU incidence (relative risk 0.37, 95% CI 0.14-1.01; *P*=.05) [53]. Nonetheless, the study showed that there was a substantial degree of variability in the studies, with 91% heterogeneity [53]. Thus, multimedia aids may not offer additional benefits beyond usual care in reducing the incidence of DFU among individuals at a moderate-high risk of DFUs, suggesting the need for more targeted educational approaches. Although our study did not find a significant effect on self-care behavior, this should not be interpreted as evidence against the long-term effectiveness of all media-based tools, which may require extended follow-ups, greater interactivity, or more tailored content to achieve meaningful and sustained behavioral change. In addition, future studies should consider evaluating the effectiveness of media-based educational tools in lower-risk populations, where such interventions may have a greater impact on preventive behaviors and outcomes.

### Strengths and Limitations

The strength of this pilot study is the long-term duration of the intervention at 12 months. However, a major limitation of this study is the unexpected COVID-19 global pandemic and associated government restrictions, which significantly impacted study conduct and led to lower enrollment and higher dropout rates than expected. This was potentially because, as a high-risk group for COVID-19, participants were reluctant to increase their contact with others unnecessarily. Although there was a higher dropout among participants with a high risk, particularly in the control group, our ITT analysis yielded results consistent with the PP analysis, suggesting that differential attrition did not substantially bias the study findings. The small sample size limits the generalizability of our findings, and thus, future studies with larger and more diverse populations are needed to validate these results and better evaluate the effectiveness of app-based interventions. Another limitation is that the MyU app lacked interactive features, which may have reduced its appeal—particularly among older participants [54,55]—although this was not formally assessed through usability testing or structured feedback during the study.

### Conclusions and Future Directions

In conclusion, the study findings revealed that a multimedia education approach alone to improve foot self-care knowledge and behavior was unfeasible and not engaging for long-term use in people at a moderate-high DFU risk. However, the reinforcement of educational material in the follow-up podiatry visit might be effective in achieving persistent changes in foot care behavior of this patient group. Given the scope of this study, further confirmative RCT studies, with a larger sample size, are needed.

## Supplementary material

10.2196/78261 Checklist 1 CONSORT-EHEALTH checklist.

## Abbreviations

CONSORT-EHEALTH: Consolidated Standards of Reporting Trials of Electronic and Mobile Health Applications and Online Telehealth
DFU: diabetic foot ulceration
HbA_1c_: hemoglobin A_1c_
ITT: intention-to-treat
NICE: National Institute for Health and Care Excellence
PP: per-protocol
RCT: randomized controlled trial
SDSCA: Summary of Diabetes Self-Care Activities

## References

[1] Ong KL, Stafford LK, McLaughlin SA (2023). Global, regional, and national burden of diabetes from 1990 to 2021, with projections of prevalence to 2050: a systematic analysis for the Global Burden of Disease Study 2021. Lancet.

[2] Magliano DJ, Boyko EJ (2021). IDF DIABETES ATLAS.

[3] (2016). State of the nation (England 2016): time to take control of diabetes. Diabetes UK.

[4] Reiber GE, Vileikyte L, Boyko EJ (1999). Causal pathways for incident lower-extremity ulcers in patients with diabetes from two settings. Diabetes Care.

[5] Abbott CA, Garrow AP, Carrington AL (2005). Foot ulcer risk is lower in South-Asian and African-Caribbean compared with European diabetic patients in the U.K.: the North-West diabetes foot care study. Diabetes Care.

[6] Singh N, Armstrong DG, Lipsky BA (2005). Preventing foot ulcers in patients with diabetes. JAMA.

[7] Boulton AJM (2008). The diabetic foot: grand overview, epidemiology and pathogenesis. Diabetes Metab Res Rev.

[8] Pecoraro RE, Reiber GE, Burgess EM (1990). Pathways to diabetic limb amputation. Basis for prevention. Diabetes Care.

[9] Siitonen OI, Niskanen LK, Laakso M, Siitonen JT, Pyörälä K (1993). Lower-extremity amputations in diabetic and nondiabetic patients. A population-based study in eastern Finland. Diabetes Care.

[10] Trautner C, Haastert B, Giani G, Berger M (1996). Incidence of lower limb amputations and diabetes. Diabetes Care.

[11] Armstrong DG, Swerdlow MA, Armstrong AA, Conte MS, Padula WV, Bus SA (2020). Five year mortality and direct costs of care for people with diabetic foot complications are comparable to cancer. J Foot Ankle Res.

[12] Lim TS, Finlayson A, Thorpe JM (2006). Outcomes of a contemporary amputation series. ANZ J Surg.

[13] Schaper NC, van Netten JJ, Apelqvist J (2020). Practical guidelines on the prevention and management of diabetic foot disease (IWGDF 2019 update). Diabetes Metab Res Rev.

[14] Ezzatvar Y, García-Hermoso A (2023). Global estimates of diabetes-related amputations incidence in 2010-2020: a systematic review and meta-analysis. Diabetes Res Clin Pract.

[15] Raju BN, Mateti UV, Mohan R (2022). Educational interventions and its impact on the treatment outcomes of diabetic foot ulcer patients. J Diabetol.

[16] Yıldırım Ayaz E, Dincer B, Oğuz A (2022). The effect of foot care education for patients with diabetes on knowledge, self-efficacy and behavior: systematic review and meta-analysis. Int J Low Extrem Wounds.

[17] Saltar L, Sahar J (2020). The intervention of foot care education in the prevention of diabetic foot ulcers: a literature review. https://proceedings.ums.ac.id/iseth/article/view/1276.

[18] Sampson EO, Abdul Manaf R, Ismail S, Shahar HK, Udeani TK (2023). Improving foot self-care practices through health education intervention programs among diabetic patients: a systematic review. Malays J Med Health Sci.

[19] Dorresteijn JAN, Kriegsman DMW, Assendelft WJJ, Valk GD (2014). Patient education for preventing diabetic foot ulceration. Cochrane Database Syst Rev.

[20] Campbell FA, Goldman BD, Boccia ML, Skinner M (2004). The effect of format modifications and reading comprehension on recall of informed consent information by low-income parents: a comparison of print, video, and computer-based presentations. Patient Educ Couns.

[21] Murphy PW, Chesson AL, Walker L, Arnold CL, Chesson LM (2000). Comparing the effectiveness of video and written material for improving knowledge among sleep disorders clinic patients with limited literacy skills. South Med J.

[22] Hoogeveen RC, Dorresteijn JAN, Kriegsman DMW, Valk GD (2015). Complex interventions for preventing diabetic foot ulceration. Cochrane Database Syst Rev.

[23] Pratiwi LD, Haryanto J, Wahyudi AS (2023). The effect of foot self care and diabetes self-management mobile application in preventing foot ulcer recurrence: a systematic review study. Malays J Med Health Sci.

[24] Obilor HN, Achore M, Woo K (2022). Use of information communication technology tools in diabetic foot ulcer prevention programs: a scoping review. Can J Diabetes.

[25] Bus SA, van Netten JJ (2016). A shift in priority in diabetic foot care and research: 75% of foot ulcers are preventable. Diabetes Metab Res Rev.

[26] (2016). Process of translation and adaptation of instruments. World Health Organization.

[27] (2015). Diabetic foot problems: prevention and management. National Institute for Health and Care Excellence.

[28] Management of diabetes: a national clinical guideline. Scottish Intercollegiate Guidelines Network.

[29] American Diabetes Association (2018). Microvascular complications and foot care: standards of medical care in diabetes—2018. Diabetes Care.

[30] Sheridan SL, Viera AJ, Krantz MJ (2010). The effect of giving global coronary risk information to adults: a systematic review. Arch Intern Med.

[31] Pollock RD, Unwin NC, Connolly V (2004). Knowledge and practice of foot care in people with diabetes. Diabetes Res Clin Pract.

[32] Rheeder P, Venn M, de Korte E, van Zyl D (2008). Knowledge of foot care in people with diabetes in a tertiary care setting. J Endocrinol Metab Diabetes S Afr.

[33] Toobert DJ, Hampson SE, Glasgow RE (2000). The summary of diabetes self-care activities measure: results from 7 studies and a revised scale. Diabetes Care.

[34] AlJohani KA, Kendall GE, Snider PD (2016). Psychometric evaluation of the Summary of Diabetes Self-Care Activities-Arabic (SDSCA-Arabic): translation and analysis process. J Transcult Nurs.

[35] Baba M, Duff J, Foley L, Davis WA, Davis TME (2015). A comparison of two methods of foot health education: the Fremantle Diabetes Study Phase II. Prim Care Diabetes.

[36] Schaper NC, van Netten JJ, Apelqvist J (2020). Practical guidelines on the prevention and management of diabetic foot disease (IWGDF 2019 update). Diabetes Metab Res Rev.

[37] Jeffcoate WJ, Vileikyte L, Boyko EJ, Armstrong DG, Boulton AJM (2018). Current challenges and opportunities in the prevention and management of diabetic foot ulcers. Diabetes Care.

[38] Kustini K, Sari Y (2023). Use of mobile applications in increasing knowledge of diabetes mellitus foot care. J Med Health Stud.

[39] Rahaman HS, Jyotsna VP, Sreenivas V, Krishnan A, Tandon N (2018). Effectiveness of a patient education module on diabetic foot care in outpatient setting: an open-label randomized controlled study. Indian J Endocrinol Metab.

[40] Beiranvand S, Fayazi S, Asadizaker M (2015). Effect of educational programs on the knowledge, attitude, and practice of foot care in patients with diabetes. Jundishapur J Chronic Dis Care.

[41] Kilic M, Karadağ A (2020). Developing and evaluating a mobile foot care application for persons with diabetes mellitus: a randomized pilot study. Wound Manag Prev.

[42] Guo SHM, Chang HK, Lin CY (2015). Impact of mobile diabetes self-care system on patients’ knowledge, behavior and efficacy. Comput Ind.

[43] Dincer B, Bahçecik N (2021). The effect of a mobile application on the foot care of individuals with type 2 diabetes: a randomised controlled study. Health Educ J.

[44] Sadler S, Gerrard J, Searle A (2023). The use of mHealth apps for the assessment and management of diabetes-related foot health outcomes: systematic review. J Med Internet Res.

[45] Firdaus M, Jittanoon P, Boonyasopun U, Che Hasan MK (2023). The effect of mHealth program on behavior modification and health outcomes among patients with diabetes: a randomized controlled trial study. Belitung Nurs J.

[46] Chen SM, Jo Wu CJ (2023). Investigating the effects of digital foot self-management program on enhancing self-efficacy and self-care behavior among community-dwelling older adults with type 2 diabetes: a randomized controlled trial. Digit Health.

[47] Suniyadewi NW, Nursalam N, Sufyanti Arief Y (2024). Effect of android-based mobile diabetic foot early self-assessment on diabetic foot prevention behaviors of Indonesian patients with type 2 diabetes. J Holist Nurs Midwifery.

[48] Moschonis G, Siopis G, Jung J (2023). Effectiveness, reach, uptake, and feasibility of digital health interventions for adults with type 2 diabetes: a systematic review and meta-analysis of randomised controlled trials. Lancet Digit Health.

[49] Lira JAC, Rocha ÁSC, Bezerra SMG, Nogueira PC, Santos A, Nogueira LT (2023). Effects of educational technologies on the prevention and treatment of diabetic ulcers: a systematic review and meta-analysis. Rev Lat Am Enfermagem.

[50] Monami M, Zannoni S, Gaias M, Nreu B, Marchionni N, Mannucci E (2015). Effects of a short educational program for the prevention of foot ulcers in high-risk patients: a randomized controlled trial. Int J Endocrinol.

[51] Pratama K, Fahrain J (2023). Prevention strategy for ulcer recurrence in patients with type II diabetes mellitus: a quasi-experimental study. Iran J Nurs Midwifery Res.

[52] Bharat Kotru SK (2025). Intervention of diabetes foot care practices on the prevention of new diabetic foot ulcers in patients with type 2 diabetes mellitus. J Diabetes Metab.

[53] Adiewere P, Gillis RB, Imran Jiwani S, Meal A, Shaw I, Adams GG (2018). A systematic review and meta-analysis of patient education in preventing and reducing the incidence or recurrence of adult diabetes foot ulcers (DFU). Heliyon.

[54] Steinert A, Eicher C, Haesner M, Steinhagen-Thiessen E (2020). Effects of a long-term smartphone-based self-monitoring intervention in patients with lipid metabolism disorders. Assist Technol.

[55] Su J, Dugas M, Guo X, Gao GG (2020). Influence of personality on mHealth use in patients with diabetes: prospective pilot study. JMIR mHealth mHealth.

